# Genome wide association analysis of the 16th QTL- MAS Workshop dataset using the Random Forest machine learning approach

**DOI:** 10.1186/1753-6561-8-S5-S4

**Published:** 2014-10-07

**Authors:** Giulietta Minozzi, Andrea Pedretti, Stefano Biffani, Ezequiel Luis Nicolazzi, Alessandra Stella

**Affiliations:** 1 Parco Tecnologico Padano Srl, Via Einstein 26900 Lodi, Italy; 2 IBBA-CNR, Via Einstein 26900, 26900 Lodi, Italy; 3 Department of Veterinary Science and Public Health, University of Milan, Via Celoria 10, 20133 Milan, Italy

## Abstract

**Background:**

Genome wide association studies are now widely used in the livestock sector to estimate the association among single nucleotide polymorphisms (SNPs) distributed across the whole genome and one or more trait. As computational power increases, the use of machine learning techniques to analyze large genome wide datasets becomes possible.

**Methods:**

The objective of this study was to identify SNPs associated with the three traits simulated in the 16th MAS-QTL workshop dataset using the Random Forest (RF) approach. The approach was applied to single and multiple trait estimated breeding values, and on yield deviations and to compare them with the results of the GRAMMAR-CG method.

**Results:**

The two QTL mapping methods used, GRAMMAR-CG and RF, were successful in identifying the main QTLs for trait 1 on chromosomes 1 and 4, for trait 2 on chromosomes 1, 4 and 5 and for trait 3 on chromosomes 1, 2 and 3.

**Conclusions:**

The results of the RF approach were confirmed by the GRAMMAR-CG method and validated by the effective QTL position, even if their approach to unravel cryptic genetic structure is different. Furthermore, both methods showed complementary findings. However, when the variance explained by the QTL is low, they both failed to detect significant associations.

## Background

Genome wide association studies (GWAs) are now widely used in the livestock sector to estimate the association among multiple single nucleotide polymorphisms (SNPs) distributed across the whole genome and one or more trait. GWAs are typically carried out on a single-point by performing a marginal chi-square test or regression. However, these methods do not take into account linkage disequilibrium between markers and the genetic structure of the population that may have a large impact on structured populations (e.g. cattle populations). Approaches for genome wide pedigree-based quantitative trait loci (QTL) analysis have been developed (*e.g*. GRAMMAR-CG), which are based on mixed model and regression, where the genomic kinship matrix estimated through genomic marker data can be used to correct for familiar correlation and cryptic relatedness [1].

As computational power increases, the use of more advanced machine learning techniques to analyze large genome wide datasets becomes possible [2], these techniques include Support Vector Machines [3], Bayesian Networks [4] and Random Forest [5].

The Random Forests (RF) algorithm [6] is a machine-learning method that has been widely applied to classification and regression problems, and is particularly well suited to circumstances in which the number of potential explanatory variables exceeds the number of observations, as is the case for GWAs. The RF algorithm produces a collection of trees (forest), each grown on a different bootstrap sample of observations, and at each split (node) of a tree, a different random subset of predictors (SNP) is evaluated to identify the best split. The final scores are then calculated by aggregating predictions resulting from all the trees grown in the forest.

RF embraces a combination of characteristics that makes it appropriate for genetic applications: it is well suited for very large datasets; it is non-parametric, thus does not require a causal model to be specified, it is highly parallelizable and considers interactions between predictors.

The objective of this study was to identify SNPs associated to the three traits simulated in the 16th MAS-QTL workshop dataset using the Random Forest approach and to compare them with the results obtained by the Grammar-CG method. SNPs identified by both methods were verified with the actual QTL positions.

## Methods

### Dataset

The dataset used was provided by the organisers of the 16th QTLMAS workshop and consisted of 4080 individuals (G0 to G4). The simulated genome was 499.750 Mb consisting of 5 chromosomes carrying 2,000 equally distributed SNPs. The GWA analysis was conducted on 3000 samples, all females belonging to generations G1 to G3, for which phenotypic information for three traits (yield deviations) was provided. The analysis was performed on: yield deviations (YD1, YD2 and YD3), the estimated breeding values (EBV) obtained from a single trait model (tr1_ST, tr2_ST, tr3_ST) and the EBVs obtained from a multiple trait model (tr1_MT, tr2_MT, tr3_MT).

### Analysis

#### Variance components and EBV estimation

Variance components and EBVs were obtained separately, using REMLF90 and BLUPF90 programs, respectively [7]. The model used to estimate variance components and EBVs was:

$$y_{k,i,j}=\mu_{k}+GEN_{k,i}+Animal_{k,j}+e_{k,i,j}$$

where *μ *is a general mean for the *k*^th ^trait, *GEN *is a fixed effect for i generations (*i *= 1 to 3), *Animal *is a random animal effect with distribution ~ N(0,σ^2^_a_), where σ^2^_a _is the additive genetic variance, and *e *is the random residual with distribution ~ N(0, σ^2^_e_), where σ^2^_e _is the residual variance. Covariance between traits was considered only in multiple-trait analysis.

### Random Forest

Feature selection (SNPs) analysis was performed with the *randomForest *package in R [8] using 3000 individuals and the 9042 SNPs that passed quality control checks out of the total 10000 SNP. The minimum size of the terminal nodes was set to 5. The number of trees grown was set to 1000. The subset of samples evaluated at each tree was 70% of the total number of samples (n = 2100). The number of variables evaluated at each node was set to the square root of the number of predictors (p = 94). All SNPs were ordered by Mean decrease Gini index [6] and the most strongly associated SNPs are at the top of the lists shown in Table 2, 3 and 4.

### Grammar-CG

Genome-wide association analysis was performed with the GenABEL package in R using a three step GRAMMAR-CG (Genome wide Association using Mixed Model and Regression - Genomic Control) approach [1,9].

## Results and discussion

### Variance components and EBV estimation

Mean and standard deviations of the nine phenotypes used are shown in Table 1. The heritability estimates resulting from the single trait model were 0.38, 0.38 and 0.50 for trait 1, 2 and 3, respectively. Large genetic correlations between traits 1 and 2 were observed (0.83), whereas lower genetic correlation was observed for trait 2 and 3 (0.12). Negative correlation was observed between traits 1 and 3 (-0.44).

**Table 1:** Statistics of the nine phenotypes used in the GWAs.

Trait	n	Mean	Sd
YD1	3000	0	176,519
YD2	3000	0	9,512
YD3	3000	0	0,024
tr1_MT	3000	-0,238	81,495
tr2_MT	3000	0,031	4,264
tr3_MT	3000	0	0,015
tr1_ST	3000	-0,555	79,057
tr2_ST	3000	0,0041	4,254
tr3_ST	3000	0	0,014

Yield deviations for the three traits (YD1, YD2 and YD3), estimated breeding values (EBV) obtained from a single trait model (tr1_ST, tr2_ST, tr3_ST) and from a multiple trait model (tr1_MT, tr2_MT, tr3_MT).

**Table 2:** Top SNPs identified by the Random Forest and GRAMMAR-CG Approach for Trait 1

			Trait 1				
**Random Forest method **					**Grammar_CG**		

**Multiple Trait EBV **				**Multiple Trait EBV **			

**SNPname**	**CHR**	**pos Kb **		**name**	**CHR**	**pos Kb **	**p-value**
SNP6499	4	24.900		SNP6499	4	24.900	3,6E-17
SNP4688	3	34.350		SNP1682	1	84.050	1,2E-07
SNP4674	3	33.650		SNP1683	1	84.100	4,4E-06
SNP4197	3	9.800		SNP6498	4	24.850	7,2E-06
SNP7145	4	57.200		SNP3585	2	79.200	1,1E-05
SNP1012	1	50.550		SNP6501	4	25.000	1,1E-05
SNP1614	1	80.650		SNP6469	4	23.400	8,4E-05
SNP6534	4	26.650		SNP9362	5	68.050	9,5E-05
**Single Trait EBV **				**Single trait EBV**			
SNP6499	4	24.900		SNP6499	4	24.900	9,8E-16
SNP4688	3	34.350		SNP1682	1	84.050	9,8E-06
SNP4674	3	33.650		SNP6501	4	25.000	1,4E-05
SNP4197	3	9.800		SNP6498	4	24.850	3,0E-05
SNP1012	1	50.550		SNP1683	1	84.100	5,0E-05
SNP1614	1	80.650		SNP293	1	14.600	5,6E-05
**Yield Deviation **				**Yield Deviation**			
SNP6499	4	24.900		SNP6499	4	24.900	1,9E-19
SNP1683	1	84.100		SNP1682	1	84.050	4,2E-09
SNP6507	4	25.300		SNP1683	1	84.100	2,8E-08
SNP1614	1	80.650		SNP6498	4	24.850	2,7E-07
SNP6506	4	25.250		SNP6501	4	25.000	6,9E-07
SNP4674	3	33.650		SNP6506	4	25.250	3,3E-06
SNP1682	1	84.050		SNP293	1	14.600	9,2E-06
SNP9374	5	68.650		SNP6507	4	25.300	2,7E-05
SNP1012	1	50.550		SNP1699	1	84.900	5,1E-05
SNP1685	1	84.200		SNP1161	1	58.000	7,6E-05

**Table 3:** Top SNPs identified by the Random Forest and GRAMMAR-CG Approach for trait 2

			Trait 2				
**Random Forest method **					**Grammar_CG**		

**Multiple Trait EBV **				**Multiple Trait EBV **			

**SNP name**	**CHR**	**pos Kb **		**SNP name**	**CHR**	**Pos Kb **	**p-value**

SNP6499	4	24.900		SNP6499	4	24.900	1,93E-18
SNP7151	4	57.500		SNP293	1	14.600	3,51E-10
SNP298	1	14.850		SNP4044	3	2.150	6,38E-10
SNP2171	2	8.500		SNP298	1	14.850	4,07E-07
SNP293	1	14.600		SNP6501	4	25.000	1,79E-06
SNP9528	5	76.350		SNP6498	4	24.850	1,74E-05
SNP296	1	14.750		SNP296	1	14.750	8,60E-05
**Single Trait EBV **				**Single Trait EBV **			
SNP6499	4	24.900		SNP6499	4	24.900	1,93E-19
SNP7151	4	57.500		SNP293	1	14.600	1,74E-09
SNP2171	2	8.500		SNP4044	3	2.150	1,24E-08
SNP298	1	14.850		SNP298	1	14.850	1,12E-06
SNP293	1	14.600		SNP6501	4	25.000	1,17E-06
SNP9528	5	76.350		SNP6498	4	24.850	8,61E-06
**Yield Deviation **				**Yield Deviation **			
SNP6499	4	24.900		SNP6499	4	24.900	2,90E-24
SNP293	1	14.600		SNP293	1	14.600	1,21E-11
SNP298	1	14.850		SNP6501	4	25.000	2,34E-08
SNP296	1	14.750		SNP298	1	14.850	4,93E-08
SNP6507	4	25.300		SNP6498	4	24.850	7,76E-08
SNP6506	4	25.250		SNP4044	3	2.150	3,19E-07
SNP6425	4	21.200		SNP296	1	14.750	1,58E-06
SNP9374	5	68.650		SNP6506	4	25.250	2,80E-06
SNP295	1	14.700		SNP295	1	14.700	3,81E-06
SNP7151	4	57.500		SNP6503	4	25.100	1,66E-05
				SNP6507	4	25.300	2,37E-05
				SNP6504	4	25.150	5,45E-05
				SNP6502	4	25.050	8,14E-05
				SNP9362	5	68.050	8,14E-05

**Table 4:** Top SNPs identified by the Random Forest and GRAMMAR-CG Approach for trait 3

			Trait 3				
**Multiple Trait EBV **				**Multiple Trait EBV **			

**SNP name**	**CHR**	**Pos Kb **		**SNP name**	**CHR**	**Pos Kb **	

SNP4738	3	36.850		SNP3585	2	79.200	1,54E-22
SNP3585	2	79.200		SNP4738	3	36.850	1,71E-14
SNP1683	1	84.100		SNP4044	3	2.150	1,52E-13
SNP3584	2	79.150		SNP1682	1	84.050	5,52E-13
SNP1291	1	64.500		SNP1683	1	84.100	7,06E-11
SNP1478	1	73.850		SNP3584	2	79.150	9,54E-11
SNP1682	1	84.050		SNP1699	1	84.900	8,86E-08
SNP1169	1	58.400		SNP1166	1	58.250	3,33E-06
**Single Trait EBV **				**Single Trait EBV **			
**RanFoG**		**Trait 3 ST **		**GRAMMAR**		**Trait 3 ST **	
SNP1683	1	84.100		SNP1682	1	84.050	7,61E-13
SNP4738	3	36.850		SNP1683	1	84.100	1,12E-12
SNP7012	4	50.550		SNP3585	2	79.200	1,36E-11
SNP1291	1	64.500		SNP4044	3	2.150	9,75E-09
SNP1169	1	58.400		SNP1699	1	84.900	2,41E-08
SNP1478	1	73.850		SNP1161	1	58.000	1,61E-07
SNP296	1	14.750		SNP4738	3	36.850	2,28E-06
SNP3585	2	79.200		SNP1178	1	58.850	1,43E-05
SNP4317	3	15.800		SNP4047	3	2.300	2,39E-05
SNP295	1	14.700		SNP3584	2	79.150	2,80E-05
**Yield Deviation **				**Yield Deviation**			
SNP1683	1	84.100		SNP1682	1	84.050	2,49E-19
SNP1682	1	84.050		SNP1683	1	84.100	2,09E-18
SNP4738	3	36.850		SNP3585	2	79.200	5,84E-15
SNP3585	2	79.200		SNP1699	1	84.900	3,29E-12
SNP295	1	14.700		SNP4044	3	2.150	3,88E-08
SNP1161	1	58.000		SNP1161	1	58.000	6,46E-08
SNP1169	1	58.400		SNP3584	2	79.150	8,54E-08
SNP296	1	14.750		SNP4738	3	36.850	2,30E-07
SNP278	1	13.850		SNP1166	1	58.250	1,86E-06
SNP1166	1	58.250		SNP1697	1	84.800	1,92E-06
				SNP1178	1	58.850	3,32E-06
				SNP1168	1	58.350	7,79E-06
				SNP1685	1	84.200	1,42E-05
				SNP3595	2	79.700	2,08E-05
				SNP4047	3	2.300	3,11E-05

### Association mapping

The two QTL mapping methods used, GRAMMAR-CG and RF, were successful in identified the largest QTLs for trait 1 on chromosomes 4 and 1 in position 24 Mb and 84 Mb, for trait 2 on chromosomes 4, 5 and 1 in position24 Mb, 68 Mb and 14 Mb and for trait 3 on chromosomes 1, 2 and 3 at 84 Mb, 79 Mb and 36 Mb respectively. Positions and names of the significant SNPs are shown in Tables 2, 3 and 4.

Both methods showed good precision in the identification of the QTL in comparison with the "real" QTL position provided by [10]. Interestingly the exact markers flankingthe QTL were identified for all traits.

Differences however were observed depending on i) the phenotype analysed, YD, single trait EBV and Multiple trait EBV and on ii) the method used RF or GRAMMAR-CG.

With regard to Trait 1, the GRAMMAR-CG method identified 8 significant associations for multiple trait EBV, 6 for single trait EBVs and 10 for YD, only 4 of which are common between the three phenotypes. The RF approach identified the same number of markers per phenotype, but only 2 markers were in common between the methods of analysis and phenotype. The two markers identified by both approaches were the QTL which explained the largest variance, however, the other markers are all true associations and indicate that using different types of phenotypes for the same trait and different analysis methods may overlap, but may also show some differences in QTLs and positions.

Traits 2 and 3 share the same pattern as observed for trait 1. Several QTL were identified in common between phenotypes and methods but just a few were in common between analysis methods: 2 markers for trait 2 and 3 markers for trait 3. When the YD phenotype was used, a larger number of significant SNPs were detected. This may be due to the larger variability of the YD compared to the more regressed EBV phenotypes (Table 1).

Interestingly both methods failed to identify the QTLs on chromosomes 4 and 5 for Trait 3. The variance explained by the markers is low, suggesting that both methods are not able to detect QTLs which explain a small amount of variance. The RF approach, however, detect the QTL on chromosomes 5 and 3 for Trait 1.

Overall the results of the RF were confirmed by the results of the GRAMMAR-CG method and were validated by the effective positions given the QTL. Interestingly, even though the RF approach does not directly use family structure information through a relationship matrix (genomic or additive), as is the case in the GRAMMAR-CG approach, correct identification of QTL positions is achieved.

## Conclusions

In this study we proposed the use of recursive partitioning approaches such as Random Forest, as an alternative to traditional regression methods to detect the genetic loci. The results of the RF approach were consistent with those of the GRAMMAR-CG method and validated by the effective positions given for the QTL. However, when the variance explained by the QTL was low, both failed to detect a significant association.

## Competing interests

The authors declare that they have no competing interests.

## Authors' contributions

AP, GM, ELN, and SB participated in the design and performed the statistical analysis. GM and AS conceived the study, participated in its design and coordination. GM drafted the manuscript. All authors read and approved the final manuscript.

## Declarations

This work was funded by the MASTFIELD project n.1745 (Applicazione di sistemi molecolari innovativi per ilcontrollo in campo delle mastiti bovine) of the Lombardy Region (Agricultural regional research programme2010-2012) and by the PON EPISUD project n° PON01_ 01841 project funded by MIUR (Ministero dell'Istruzione, dell'Università e della Ricerca).

This article has been published as part of *BMC Proceedings *Volume 8 Supplement 5, 2014: Proceedings of the 16th European Workshop on QTL Mapping and Marker Assisted Selection (QTL-MAS). The full contents of the supplement are available online at http://www.biomedcentral.com/bmcproc/supplements/8/S5

## References

[B1] Amin N van Duijn CM Aulchenko YS A genomic background based method for association analysis in related individuals PLoS ONE 2007 2 e1274 18060068 10.1371/journal.pone.0001274 PMC2093991

[B2] Goldstein BA Polley EC Briggs FB Random forests for genetic association studies Stat Appl Genet Mol Biol 2011 10 1 32 22889876 10.2202/1544-6115.1691 PMC3154091

[B3] Yoon Y Song J Hong S Kim J Analysis of multiple single nucleotide polymorphisms of candidate genes related to coronary heart disease susceptibility by using support vector machines Clin Chem Lab Med 2003 41 529 534 12747598 10.1515/CCLM.2003.080

[B4] Han B Chen XW Talebizadeh Z Xu H Genetic studies of complex human diseases: characterizing SNP-disease associations using Bayesian networks BMC Syst Biol 2012 6 23281790 10.1186/1752-0509-6-S3-S14 PMC3524021

[B5] Mokry FB Higa RH de Alv arenga Mudadu M Oliveira de Lima A Meirelles SL Barbosa da Silva MV Cardoso FF Morgado de Oliveira M Urbinati I Méo Niciura SC Tullio RR Mello de Alencar M Correia de Almeida Regitano L Genome-wide association study for backfat thickness in Canchim beef cattle using Random Forest approach BMC Genet 2013 14 47 Jun 5 23738659 10.1186/1471-2156-14-47 PMC3680339

[B6] Breiman L 2001 Random Forests Machine Learning 2001 45 5 32

[B7] Misztal I Tsuruta S Strabel T Auvray B Druet T Lee DH BLUPF90 and related programs (BGF90) Proc. 7th WCGALP Montpellier 2002, France Communication No. 28-07

[B8] http://cran.r-project.org/web/packages/randomForest/index.html

[B9] Aulchenko YS Ripke S Isaacs A van Duijn CM GenABEL: an R package for genome-wide association analysis Bioinformatics 2007 23 1294 6 17384015 10.1093/bioinformatics/btm108

[B10] http://qtl-mas-2012.kassiopeagroup.com/en/dataset.php

